# Some Observations on the Present Epidemic Dysentery

**Published:** 1781-12

**Authors:** 


II. *Some observations on the present epidemic dysentery.* By Francis Geach, *Surgeon of the Royal Hospital at Plymouth, and Fellow of the*

*Royal Society.* 8vo. Baldwin, *London*, 1781. 41 pages, 1s.

WE have here an account of a dyſenteric fever that prevailed in Plymouth in September 1781.

The author obſerves, that it began with the uſual ſymptoms of ſickneſs at the ſtomach, anxiety at the *præcordia*, and griping in the belly. Theſe ſymptoms were ſoon followed by bloody ſtools and mucus, in great quantities, with a conſtant teneſmus. The pulſe in ſome patients at this time was hard and full; in others remarkably low and creeping, attended ſometimes with a pungent heat of the ſkin, and often with partial and unprofitable ſweats. The tongue was uſually coated with a bilious mucus; the abdomen was tenſe, and in ſome caſes there was a ſuppreſſion of urine, or at leaſt ſome difficulty in voiding it. As the diſorder advanced, the diſcharge from the inteſtines became more and more bloody, the mucus in greater quantity, accompanied with much fœtor, and at laſt by the ſmell and ſymptoms it might be ſuppoſed, that a mortification had taken place. What appearances there were after death, the author is unable to ſay, having had no opportunity to examine any deceaſed ſubject.

Speaking of the ſtate of the weather at Plymouth previous to the appearance of the epidemic, Mr. Geach remarks, that the ſummers for ſome years paſt have been much warmer and drier than uſual, and that although the dyſentery has appeared more or leſs towards the autumnal equinox, it was till now chiefly confined to the troops in the barracks, whereas this year it became in a manner univerſal; for although it began with perſons of a relaxed habit and children, yet at laſt perſons of all ages and conditions were ſeized with it. Neither was there much difference obſerved, whether they were abſtemious or not as to any particular food or drink. New unfermented cyder, the commonly alledged cauſe of the dry colic and dyſentery at this ſeaſon in other years, could not be adduced now, as there was hardly any cyder made. Raw oyſters, however, we are told, eaten ſoon after their being taken out of the ſea, almoſt univerſally brought a flux on the eaters. Many of the oyſters indeed were as yet milky, and not come to their due ſtate of firmneſs and healthineſs.

The ſymptoms and progreſs of the diſeaſe were generally much the ſame; but it was obſerved, that thoſe who lived in large, clean, and airy neither ſuffered ſo much, nor were ſo ſoon infected, as thoſe who were confined to low, cloſe dwellings, badly ventilated, and fuller of noxious effluvia.

Speculating with regard to the cauſe of this epidemic, our author obſerves, that "its origin was with reaſon perhaps deduced from the winds, and from the planets, or from ſubterranean vapours, ſulphureous, or other mineral effluvia thrown out into the air by earthquakes, or collected there in great abundance from long drought, from the evaporation of bogs and marſhes, which abound with phlogiſton, or a ſulphureous principle."

That there is a communication of matter between all the planets, and particularly of the electric fluid, and that this fluid may carry with it many noxious particles, he mentions as an opinion of the late Dr. Alcock, and ſpoken of in the memoirs of his life lately publiſhed; and as a proof that ſulphureous ſteams are capable of producing the dyſentery he relates the following curious fact: "It was obſerved at one time, that almoſt all the men received into the hoſpital (at Plymouth) ſoon became dyſenteric, and the flux was ſo deſtructive as to ſet all medicine, and all cure at defiance.

"This

" This circumſtance was mentioned to Dr. Max-

" well, at that time a commiſſioner of the Sick

" and Hurt Office, and then on his viſit; who

" thought at firſt this diſorder might proceed

" from the ſituation of the hoſpital, as it was

" contiguous to a large marſh liable to inunda-

" tions: but on my obſerving to him, that in

" times paſt the diſorder had not been parti-

" cularly confined to that hoſpital, this reaſon

" was given up. One day the Doctor remark-

" ing, that the wards ſmelt ſtrongly of ſulphur

" from the fumigation of bedding in an apart-

" ment connected with the rooms where the

" ſick lay, he ordered the fumigation to be

" diſcontinued; and the flux thereupon entirely

" ceaſed." To this paſſage the author has added the following note: " A young man at

" Tamerton, by a long and large uſe of the

" *Flos Sulphuris*, fell into a dreadful dyſentery.

" The blood was ſo broken, that he bled at

" the caruncles, and died altogether putrid."

The method of cure that ſucceeded beſt with our author in this diſeaſe was as follows: When he was called in early, the patient, if the pulſe were full, was bled; but he never ſaw, except in one inſtance, the blood buffy and tenacious.

Then,

Then, whether the patient was bled or not, a vomit of ipecacoanha was given, and the robuſt were purged with four grains of calomel and thirty grains of rhubarb. No attention was paid to irritation, nor laudanum given, to take off the ſpaſms, as he always obſerved that the irritation and mucus ceaſed at once upon a due diſcharge of fæces; the deſcent of which ſhewed that the diſorder was in a fair way of being ſubdued. For during the violence of the complaint, much blood and mucus were voided, with little or nothing excrementitious. It was alſo remarkable, that no fæces came down, till the mucous coat on the tongue began to clear away from the ſides of it; nay in ſome caſes not till the whole tongue became clean: and this obſervation, we are told, held ſo univerſally true, that it might be predicted, almoſt to a certainty, that as ſoon as the mucus on the tongue began to ſeparate and diſappear; the fæces would ſoon deſcend, and the diſorder relent. Every token of amendment then followed. The pulſe became calm and open, the eyes clear, the ſkin ſoft, the perſpiration equable. If the firſt doſe of calomel did not procure a diſcharge (and the abovementioned firſt doſe did not always ſucceed) rhubarb to the quantity of thirty grains grains was repeated, together with an ounce of ſpirituous tincture of rhubarb; and theſe, together with two grains of emetic tartar in eight ounces of water, were taken daily till the end was anſwered, the emetic tartar being uſed gradually, in ſmall quantities. Mr. Geach has ſeen, however, more than once, that a violent vomiting from the uſe of this medicine or ipecacoanha removed the complaint at once, tho' blood and mucus had juſt before been diſcharged in great quantities.

The patient was allowed to drink freely of tamarind decoction, and to ſip mutton broth without a ſcrupulous regard to the fever, but was not allowed panada, water gruel, or any thing that might tend to inflation.

In ſome inſtances, after the emetic had been given, the vomiting continued, and large quantities of a porraceous fluid were thrown up. Under this circumſtance nothing ſucceeded ſo well as calomel and rhubarb, together with the abſorbents, *magneſia*, *teſtacea*, &c. and this porraceous vomiting always ceaſed on the removal of the inteſtinal obſtruction.

The author obſerves, that the fever ſeemed to claim moſt attention, and that when this was removed, the bloody ſtools, the mucus, and of courſe the teneſmus gave way, ſo that it was ſeldom neceſſary to give opiates or aſtringents, unleſs a diarrhœa ſupervened, and continued long after the dyſentery was at an end. When this happened, lime water mixed with milk proved very efficacious. But even here he tells us, the utmoſt caution was required, for inflation of the belly ſometimes followed; and as often as this occurred, there was a neceſſity again to adminiſter an opening medicine. And, it is worthy of remark, that very often, though the dyſentery had run out to a great length, though the fæces had come down, yet when the belly was inflated after the uſe of aſtringents, the tongue would again become white, though not coated. In ſhort, while this whiteneſs remained, whether aſtringents had been uſed or not, if the patient had ſtill much pain, and the fever did but little abate, there was reaſon to ſuſpect that indurated fæces were ſtill behind, and Mr. Geach has ſeen large ſcybals actually expelled (after a fortnight's illneſs) to the ſpeedy recovery of the patient.

As the diſorder attacked every one nearly in a ſimilar manner, it yielded to the ſame mode of treatment. Tender and delicate women were found to require much the ſame medicine and proceſs, as the more robuſt. Women, who gave gave fuck, were enjoined to keep their infants at the breaft, and when this injunction was not followed by the mother, fhe felt an inconvenience. Her face flufhed, and her head became difordered by an augmentation of fever. Neither did the infants themfelves fuffer by fuch maternal indulgence; for our author never obferved that the infection, which feized on the mother and her attendants, ever affected the child in confequence of her giving fuck. Infection came from without, another way. To guard againft this infection vinegar was kept boiling in the chambers of the fick, or the bark of Cafcarilla was burnt, which in a great meafure corrected the horrible fœtor ufually attendant on the advanced ftage of the diforder.

It is remarked by our author, that the dyfenteries that have prevailed at different times in the Royal Hofpital at Plymouth, have in general foon yielded to a grain or two of ipecacoanha, and half a drachm of cream of tartar, given every fixth or eighth hour, together with tamarind whey or tamarind decoction. The patients to whom thefe medicines were adminiftered, for the moft part were fcorbutic; and their fluxes were brought on (as was thought) by a fudden tranfition from falt provifions, affording too little nutriment, to a diet of fresh meat and vegetables, affording too much. But in this epidemic dysentery these means failed, and it was found that ipecacoanha was but of little use unless it operated as an emetic, and then it relieved the patient considerably. The same thing was observed of emetic tartar.

With regard to the bark and opium, recommended and given by many in this disorder, even in its first stage, Mr. Geach remarks, that the antiseptic virtue attributed to the one, and the power of calming irritation for a time existing in the other, will not, as far as he has experienced, subdue this fever and flux, but will sometimes do mischief by stopping up an evil, which, if the patient die not of the fever, will burst out elsewhere; or, if they should not have such an effect, they generally will inflate the belly, and this inflation can only be cured by a natural or artificial return of the flux, or at least a diarrhœa.

In a note to this part of the work the author informs us, that in a high degree of alkalescent scurvy cream of tartar used as a purgative, answers, in many respects, better than the bark; frequent doses of the latter given to remove that disorder, when the patients have been sallow and bloated, bloated, having caused large indurations in the glands of the neck and axilla; but by some weeks continuance of cream of tartar, such swellings have disappeared or suppurated. He has seen this effect lately in six patients received from ships into the Royal Hospital.

In treating of the diet of the sick in the epidemic dysentery, Mr. Geach observes, that few rules could be laid down. Mutton broth till the appetite returned was the chief sustenance. Some of his patients at the beginning of the disorder were eager for cyder; and cyder well fermented, and old, was given them not with any ill, but a good effect. One precaution however was observed, not to deluge their stomachs with large draughts of this or any other liquor. They were also, at their own request, indulged with beer without any prejudice.

Inspection of the stools, however disagreeable, is recommended to the practitioner, as it may often determine what medicine should be administered. On this subject Mr. Geach remarks, that when the discharge is crude and frothy, the use of a purgative in small doses is indicated, while that which is ragged, green, or watery, shews that the disorder will be either fatal or protracted to a long period.

Whatever

Whatever be the contents of the ftools, they are directed to be removed as foon as poffible, and the foul air purified by fumigation and fire, and diffipated by opening the windows, in order to prevent contagion. The fame precautions are recommended with refpect to bedding and cloathing. Negligence in thefe matters, obferves our author, may in part be productive of the diforder, and at any rate will tend to aggravate it, and fpread the infection to others.

